# Understanding the role of power and its relationship to the implementation of the polio eradication initiative in india

**DOI:** 10.3389/frhs.2022.896508

**Published:** 2022-09-06

**Authors:** Piyusha Majumdar, S. D. Gupta, D. K. Mangal, Neeraj Sharma, Anna Kalbarczyk

**Affiliations:** ^1^SD Gupta School of Public Health, IIHMR University, Jaipur, Rajasthan, India; ^2^Indian Institute of Health Management Research, Jaipur, Rajasthan, India; ^3^IIHMR University, Jaipur, Rajasthan, India; ^4^International Health, Bloomberg School of Public Health, Baltimore, MD, United States

**Keywords:** power relation, global health, implementation science, governance, implementation challenges

## Abstract

**Background:**

Power is exercised everywhere in global health, although its presence may be more apparent in some instances than others. Studying power is thus a core concern of researchers and practitioners working in health policy and systems research (HPSR), an interdisciplinary, problem-driven field focused on understanding and strengthening multilevel systems and policies. This paper aims to conduct a power analysis as mobilized by the actors involved in implementation of the polio program. It will also reflect how different power categories are exerted by actors and embedded in strategies to combat program implementation challenges while planning and executing the Global Polio Eradication Initiative.

**Methods:**

We collected quantitative and qualitative data from stakeholders who were part of the Polio universe as a part of Synthesis and Translation of Research and Innovations from the Polio Eradication Project. Key informants were main actors of the polio eradication program, both at the national and sub-national levels. Research tools were designed to explore the challenges, strategies and unintended consequences in implementing the polio eradication program in India. We utilized Moon's expanded typology of power in global governance to analyze the implementation of the polio eradication programme in India.

**Results:**

We collected 517 survey responses and conducted 25 key informant interviews. Understanding power is increasingly recognized as an essential parameter to understand global governance and health. Stakeholders involved during polio program implementation have exerted different kinds of power from structural to discursive, moral power wielded by religious leaders to institutional power, expert power used by professional doctors to commoners like female vaccinators, and network power exercised by community influencers. Hidden power was also demonstrated by powerless actors like children bringing mothers to polio booths.

**Conclusion:**

Power is not a finite resource, and it can be used, shared, or created by stakeholders and networks in multiple ways. Those people who seem to be powerless possess invisible power that can influence decision making. Moreover, these power categories are not mutually exclusive and may be deeply interconnected with each other; one type of power can be transformed into another. Power and relations play an important role in influencing the decision-making of the community and individuals. Mid-range theories of core implementation science like PARIHAS and CFIR can also add an important variable of power in their construct necessary for implementation success of any health program.

## Background

Power is exercised everywhere in global health, although its presence may be more apparent in some instances than in others. Power is a critical concept to understand and transform health policy and systems. Health systems are therefore influenced by the power dynamics that underlie societal interactions. Power manifests implicitly and explicitly in diverse ways in the interactions of health system actors at the local, national, and global levels. These interactions span the dynamics between patients and providers at primary health facilities to the negotiation between national and international level actors regarding resource distribution and health policy priorities. Indeed, power shapes health policy and practice, including community collaboration, participation and ownership, access, affordability, and quality of health services, and the prioritization and development of health policy (1). Studying power is thus a core concern of researchers and practitioners working in health policy and systems research (HPSR), an interdisciplinary, problem-driven field focused on understanding and strengthening multilevel systems and policies. Researchers conducted a literature review related to “health policy” and “systems of LMIC” and found that power is a neglected area of research work (1, 2).

**Figure 1:**
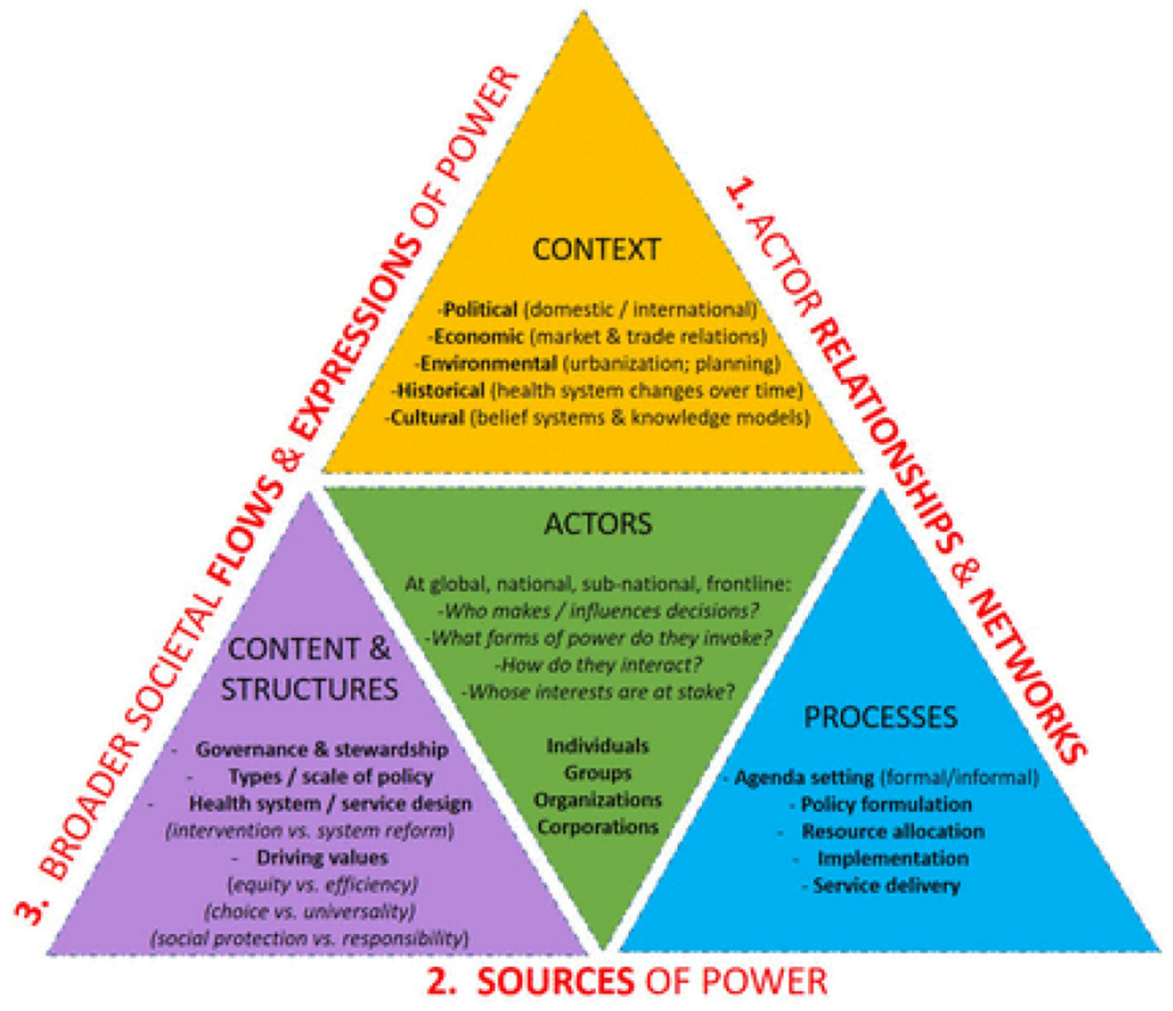
Image source: https://gh.bmj.com/content/6/11/e007268.

Power has been conceptualized in many ways including as “coercion” (3), “capital” (4), and “control” (5) and can be applied overtly or covertly *via* individuals, institutions, structures, etc. (6–8). Power is imposed, negotiated, and contested in diverse ways in health policy formulation and implementation in health system functioning. Research into power in the field of Health Policy and System Research (HPSR) generally focuses on how the expression of power enables or blocks health system change or policy implementation or what types of power are implicated in the process (2). Sriram identified six sources of power that arise in the broader social science literature: (1) technical expertise, (2) political power, (3) bureaucratic power, (4) financial power, (5) networks and access, and (6) personal attributes. In HPSR these can be mapped to Walt and Gilson's seminal “Policy Triangle” which highlights the role of the actor's relationships and network within a broader societal flow (see Figure 1). Power therefore cannot be exerted in isolation from the actor, context, content, structure, and policy of the process or system in focus (2, 9).

Each of these concepts, from the sources of power to the critical relationships between individuals, networks, and systems represent important facets of Implementation Research (IR), as highlighted by the Consolidated Framework for Implementation Research (CFIR). CFIR is comprised of five major domains (the intervention, inner and outer setting, the individuals involved, and the process by which implementation is accomplished) which interact in rich and complex ways to influence implementation effectiveness (10). The presence or absence of CFIR constructs can explain “why” implementation was or was not successful. However, to our knowledge, current IR frameworks, including CFIR, do not explicitly recognize power as dimension of implementation.

Analyzing and engaging with power can promote more transparent, equitable, and fair health systems in low- and middle-income countries (LMICs). In addition to power analysis, complementary disciplinary approaches, often from the social sciences, have been applied to describe how power affects health policymaking and health service delivery in LMICs. Anthropologists have used critical ethnography to uncover and scrutinize the power imbalances that shape the interplay between local realities (for communities and health providers) and national and global forces. And current global discourse addressing movements to decolonize global health stress the role of power and privilege more broadly in the field and call for transformational change in global health partnerships (11–14).

The research presented in this paper is part of STRIPE (Synthesis and Translation of Research and Innovation in Polio Eradication) project that collected the data from Polio stakeholders (i.e., individuals involved in the implementation of Global Polio Eradication Initiative) in India (10, 15). We conducted a power analysis mobilized by the actors involved in Polio Program Implementation to understand how power has been utilized in negotiating implementation challenges for polio eradication and to reflect on how power configuration evolved in different contexts across India. We believe this work could be used to address health equity in health systems.

## Methods

The data presented in this manuscript is from the STRIPE project (https://stripe.jhu.edu/) in India, (10) to collect information on experiences, challenges, and barriers in implementation and alternate strategies for polio eradication. The study followed a mixed-methods design which included a quantitative survey, and key-informant interviews. The study was conducted in India at the national and sub-national levels. Data collection took place August 2018 to January 2019.

### Polio universe/sample

In India, an estimated over 2.4 million people were involved in polio eradication activities. The polio universe consisted of persons from Global Polio Eradication Initiative (GPEI) partner organizations, permanent and contractual employees of central and state government, members of national and international NGOs, teaching and non-teaching staffs of schools, medical and non-medical staff of different at levels of government hospitals and frontline health workers, media persons and volunteers. In addition, influencers from religious institutions like temples, mosques, church etc. were included. We held a stakeholder consultation workshop with high level actors which enabled a list of 4,957 contacts (10, 16).

### Quantitative survey

We contacted each of these individuals who were once a part of the polio universe, approached through both online and offline survey. A self-administered online bilingual survey tool was emailed to the participants, and responses were collected on Qualtrics. Five hundred and seventeen (*n* = 517) participants (352 online and 165 offline) responded to the survey (16).

We used a purposive sampling method to recruit key informants. This sample was derived from individuals who completed the survey and identified key implementation challenges for India. Key informants included the main actors who played a pivotal part in polio eradication program at the National, state, and district levels. Informants also included change agents and frontline workers at the National, state, and district level who played a crucial role in resolving the prioritized challenges faced during the GPEI. Since the Indian government did not achieve polio elimination on its own, a collaboration between the government, non-governmental organizations, the public and private health sectors, and the general public was paramount to India's success in Polio elimination. We also interviewed change agents from implementing organizations like WHO, UNICEF, Rotary, BMGF, academic and research institutions. As per the definition of Roger, “*Change agents are people who are actively developing the skill, confidence, power, relationship and courage to make a positive difference”* (17). For our research, prominent stakeholders who played pivotal part in polio eradication program were chosen as a change agent both at national and sub-national level.

### Key informant interviews

Face to face and in-depth, semi-structured interviews were undertaken with the National change agents (*N* = 11), subnational change agents (*N* = 10), and frontline workers (*N* = 4) with a trained qualitative researcher and bilingual note-taker taking detailed notes during the interviews.

Prior to participating in the study, we read out to the potential study participants a plain language statement that outlined the study purpose and the voluntary nature of participation and obtained written informed consent. Interviews were conducted mainly in English depending on participants' preferences and in a location convenient to the participants. Each key informant interview took ~70–100 min and was recorded using a digital recorder. Data were transcribed by an experienced transcriber and checked by the interviewer.

Members of the STRIPE Team based at the Johns Hopkins Bloomberg School of Public Health developed the research tools based on CFIR and the Socio-Ecological Model (SEM). The SEM considers the complex relationships between factors that influence the individual, interpersonal, organization, community, and larger environment (10, 18).

### Analytic framework—moon's taxonomy of power

Moon's (19) expanded typology of power was built on the existing framework of Dahl's (3), Bourdieu's, and Barnett and Duvall's (6), Sriram's et al. (2) existing concept of power (8, 9).

We used Moon's expanded typology of power as our analytic framework. Moon describes eight categories of power available to actors, described in Table 1 (19). Such power, if utilized by actors, stakeholders, and institutions, can influence the thinking or action which in turn affects the decisions of other people. We explore how these taxonomies of power were exercised in the GPEI in India to combat implementation challenges and develop implementation strategies.

**Table 1:** Power category based on moon's taxonomy.

**Category of power based on moon's taxonomy**	**Description**
Physical power	Used when an actor uses or threatens to use physical force to shape the thinking or actions of other actors
Economic power	Exercised by using use of material resources (e.g., money, goods) to shape the thinking and actions of other actors
Structural	Wielded through the use of an actor's position in the structures of society to shape the thinking and/or actions of other actors
Institutional	Wielded through an actor's use of rules and decision-making procedures to shape thinking and action.
Moral	Wielded when an actor shapes the principles that others believe to be right or wrong, and the actions that may then follow
Expert	Wielded when an actor shapes what others consider to be legitimate knowledge, and therefore what they understand to be factually true or correct
Discursive	Wielded when actors shape the language others use to conceptualize, frame, and thereby define and understand an issue
Network	Wielded when individuals use their personal relationships with others to shape their thinking and/or action

## Results

### Quantitative documentation of implementation barriers

The demographics, organization representation and polio program goal and activities are mentioned in Table 2. Survey respondents worked at state, district, or sub-district level in implementing vaccination activities. Most respondents worked for government agencies and implementing partners (64%, *n* = 418), followed by GPEI partners (WHO, UNICEF, CDC, BMGF, and Rotary International) and other non-governmental organizations. Participants were asked to identify which polio eradication activities they were involved in. Majority (51%, *n* = 266) of the respondents were engaged in vaccinating children under Routine Immunization Program, providing supplementary doses of oral polio vaccine (OPV) to all children under 5 years of age during national immunization days (NIDs) and mop up activity (house to house activity where two rounds of Polio Immunization within 4–6 weeks apart conducted to limit the transmission of wild poliovirus). Around 20–23% *n* = 116–127 of the participants also involved in other activities like polio surveillance, community engagement, and monitoring and evaluation of the program. Less than 10% of respondents mentioned participating in advocacy activities, fundraising, resource mobilization, partnership or alliance development, strategy development, and delivery strengthening.

**Table 2:** Demographics of survey respondents.

**Level at which involved**	**Frequency (*N*-517)**
Global	2.47% (14)
National	7.94% (45)
State	12.7% (72)
District	25.4% (144)
Sub–district	51.5% (292)
**Organization representation**	
GPEI partners	22.5% (220)
Government/Implementing	64.2% (418)
Global NGO	2.5% (27)
Research organization	5.4% (51)
Others	3.3% (31)
**Polio program goal**	
Resource mobilization	3.4% (18)
Partnership development	4.0% (24)
Strategy development	9.4% (49)
Strengthening delivery system	12.5% (65)
Vaccination	51.2% (266)
Surveillance	24.5% (127)
Community engagement	22.4% (116)
Monitoring and evaluation	22.4% (116)

Survey respondents were able to select multiple responses for the following characteristics: the levels where they worked, their organizational representation, and the polio program goal over the period 1988–2019. Hence, the sum of responses (n) under each of these characteristics is greater than the total number of respondents (N = 517 for the survey). This sample therefore includes individuals who may have primarily worked at the global level but also supported polio eradication in one or multiple countries 2 Survey respondents were asked to indicate polio program goals which they were involved as part of the survey response; this information was not gathered during key informant interviews, though respondents' experiences were reviewed a priori to ensure representativeness across program goals.

The detailed description of implementation barriers to polio program success (As per CFIR) are presented in the Table 3. Survey respondents identified major challenges faced at the time of process of implementing the GPEI program in the field including planning, engaging, executing, reflecting and evaluating the program (*n* = 151) and the political, social, economic, technological environment (*n* = 126). Stakeholders also reported challenges with the program's characteristics (*n* = 74), Organization characteristic (n-69) and at the individual level (*n* = 44).

**Table 3:** Implementation barrier to polio program success (as per CFIR framework).

**Intervention characteristic**	**Activities conducted to enable implementation, including technologies adopted**	**Key informant interviews** ***N* (25)**	**Survey data *N* (74)**
**Construct 1: Intervention characteristics**			
Intervention source	Perception of whether the intervention was developed internally or externally led to challenges	• Imbalance between global and national priorities • Community distrust of western intervention	13.5%
Evidence	Perception of the quality and validity of the evidence did not support belief that the intervention would have the desired outcomes	• Concerns about relative effectiveness of OPV and IPV	9.4%
Relative advantage	Perception that there was another, better approach	• Concern that polio program is run in parallel to (and at expense of) routine immunization	17.5%
Adaptability	The activity was not adapted, tailored or refined to meet local needs	Lack of understanding of community norms to guide adaptation of implementation activities	20.2%
Trialability	No ability to test on a small scale and reverse course if warranted	• Perception of polio program as too big to fail even in the face of coordination and implementation failure affecting certain activities	5.4%
Complexity	Perceived difficulty of implementation reflected by its duration, scope, radicalness, disruptiveness, centrality, intricacy, and number of steps required	Perceived difficulty of implementation reflected by its duration, scope, radicalness, disruptiveness, centrality, intricacy, and number of steps required *Delivering the oral polio vaccine on time to the remotest corner has been an operation feat* Vaccine failure (VAPP) Gaps in AFP surveillance	16.2%
Cost	Cost of intervention and its implementation, including investment, supply, and opportunity costs	Difficulty financing program functions previously supported by donors • High cost of implementation in hard-to-reach areas like Kosi River in Bihar Infrastructure gaps/deficit in the interior areas in the states, lack of approachable roads, lack of infrastructure for appropriate Polio Booth	6.7%
**Organizational characteristics**	**Factors related to the organization(s) supporting implementation**	**Key informant interviews** ***N*** **(25)**	**Survey data** ***N*** **(69)**
**Construct 2: Inner setting**			
Structure	The age, social architecture, and size of an organization led to challenges	• Shifting structure of global partnership • Understaffing and shifting roles of staff	11.5%
Networks	The nature and quality of formal and informal communication within an organization led to challenges	Limited communication channels between extension workers, program leads Lack of synergy and coordination among various multiple stakeholders	20.2%
Culture	The norms, values, and operating assumptions of an organization led to challenges	• Priorities dictated by managers • Limited voice given to field workers to propose adaptations	24.6%
Implementation climate	Limited capacity for change, the receptivity of the team to the proposed intervention, the relative priority of project, organizational goals, incentive and rewards, etc. led to challenges	• Lack of consensus on program strategy • Waning prioritization of polio among some stakeholder *It is harder to have concentrated efforts in early 2000 where the immunization rounds went for 6–8 times in a year* challenges related to trust among the partners & stakeholders	23.1%
Implementation readiness	Lack of leadership engagement, limited available resources and poor access to knowledge and information led to challenges	• *MLAs, the members of parliament, the local body chairmen were involved in Polio Program and there was no major political lacking* • Chronic underfunding of the health system	17.39%
**External factors**	**Political, economic, social, technological, legal, and other environmental factors**	**Key informant interviews N (25)**	**Survey data** ***N*** **(126)**
**Construct 3: Outer setting**			
Social	Communities are non-accepting and/or resistant to the intervention	Vaccine hesitancy *There was initial resistance in some dominating population in western U.P due to myths and misinformation* • Community fatigue given repeated campaigns, misaligned priorities • *Determinants like basic Sanitation and Hygiene, availability of clean water, diarrhea* • Misinformation, Lack of Information *Polio drops are* anti-fertility vaccines that can make their son impotent *Polio vaccine has been grown in the kidney of pigs*	73.0%
Economic	Insufficient revenue sources	• Limited economic resources	11.9%
Political	Policymaker disinterest or resistance, limited windows of opportunity within the political climate, political structure non-conducive to coordinated action	• Low political will *Government Change in Uttar Pradesh 2002* • Insecurity and conflict *Naxalite affected areas in states of North-east India*	6.35%
Technological	Slow or limited advances of technologies used in implementing program activities	Maintenance of cold chain system (−20 degree) in extreme temperature	0.79%
Other	Challenges related to physical and human geography	• Geographical inaccessibility *(In Bihar, Kosi riverine area one such great example that has neither roads nor bridges and can only be accessed by boat, motorcycle, bicycle or on foot)* • Missing children for vaccination due to migration	7.94%
**Characteristics of individuals**	**Characteristics of individuals within an organization involved in polio eradication activities**	**Key informant interviews** ***N*** **(25)**	**Survey data** ***N*** **(44)**
**Construct 4: Characteristics of individuals**			
Knowledge	Knowledge and beliefs about the activity—individuals did not have positive attitude toward the program, were unfamiliar with facts, truths, and principles related to the intervention	Misconceptions about the vaccine and its effects • Lack of awareness of vaccine benefits Explain and convince those families whose child contracted vaccine derived polio	43.1%
Stage of change	How likely (or not) the individual is to provide skilled, enthusiastic and sustained support of the program throughout the different stages of implementation	Too many and too frequent vaccination resulting from campaign leads to vaccine fatigue from the communities Frequent and sporadic outbreaks lead to health system fatigue	22.7%
Perception of organization	Poor perception of the organization and degree of commitment to the organization	Target based approach and working in a vertical program affected the commitment of frontline workers	22.7%
Self-efficacy	Lack of belief in one's own abilities to execute required courses of action	• Health workers' lack of understanding of the program and building confidence Retain ongoing momentum of vaccinators	11.3%
**Process of activities**	**How activities were implemented**	**Key informant interviews** ***N*** **(25)**	**Survey data** ***N*** **(151)**
**Construct 5: Process of Implementation**			
Planning	Implementation schemes/methods not planned in advance, or poor quality of such methods	• Poor quality enumeration • Enormous size of the campaign (0.65 million polio booth & immunization of 170 million children) • Difficulty in planning large-scale changes, e.g., the switch from tOPV to bOPV	14.5%
Executing	Failing to carry out activities according to plan	• Lack of accountability mechanisms • Environmental risk factors like heat and monsoon created a perfect storm for virus transmission in Uttar Pradesh From vaccine procurement to vaccine logistics, whether the vaccine boxes had to be lifted at some railway station or airport and keeping a track of all those were encountered on a routine day to day basis and mobilizing the human resources seems to be challenging task	33.7%
Complexity (engaging)	Difficulty attracting and involving appropriate stakeholders in implementation	Difficulty identifying appropriate stakeholders to engage given diverse administrative structures, cultural norms • Community mistrust	27.1%
Reflecting & evaluating	Difficulty monitoring program progress and quality, including lack of regular debriefing about progress and experience	• Lack of supervision • Lack of formal processes for analyzing monitoring data and adapting plans accordingly Reach the remotest corner, inaccessible areas and influence them to be part of immunization schedule and comply with the whole dose schedule	23.1%

The italic indicates reaching the unreached.

Open-ended responses were also solicited in the survey. At the field level, stakeholders mentioned resistance and refusal to accept vaccination in the early part of the implementation of the program could not be forgotten—which led to the hiding of the children in the households. Initiation of supplementary immunization activities caused serious concerns to the lack of trust in vaccines or immunization program based on the belief that vaccines are a part of global conspiracies against some communities.

“*When started going house to house, people started resisting, because it has suddenly become endorsed program by the government. People got angry; they thought this was a ploy like a family planning program “do boond meant do bacheh.” So that rumors spread, and that too spread mostly in the northern states” Survey, STRIPE, India*.

“*The community was resistant. They would throw stones at us and heated oil and throw on us from the roof, and they would hide their children, especially male children because they thought that their children will get impotent, and we had to adopt one strategy after the other to win their trusts. Because every six weeks, there was a supplementary immunization activity in every week and people were very suspicious as to why they are coming again and again”—Survey STRIPE, India*.

Reasons for facing challenges during implementation was the duration of the Polio campaign; its frequency, community fatigue, stakeholders perceived that it was one program that took huge time, frequent activities, and involved a lot of human resources. Getting other health programs completely stalled during the polio campaign period was another factor. The shortage and unwillingness of vaccinators to participate in the campaign were surfaced due to less honorarium, longer period work, the demand for high-quality activity, and stringent actions for minor faults. involvement of other departments and partners for such a longer period emerged as a challenge. The program has become the victim of its success; many times, villages/communities boycotted the campaign for other developmental demands.

On asking the strategies that have contributed toward program's success, around 41.8% of the respondents stated that the process of conducting Pulse Polio immunization program (planning, execution. strategies, reflection and evaluation of activities, or adjustments made to the plan) was found to be the biggest internal contributor to implementing the polio vaccination activities. This was followed by other internal factors like characteristic of the individual involved in organizing the activities. The most frequently mentioned external contributor was changing the social environment (68.7%) in which the children were vaccinated (adapting toward the socio-cultural beliefs around immunization).

Qualitative data also explored major barriers owing to outer settings mainly social environment and how power played an important role in developing successful implementation strategies for eradicating polio.

- India's social environment acted as a barrier for implementation of pulse polio vaccination and strategies that was developed on the paradigm of social norms around immunization, engaging with the resistant communities created conducive social environment by different actors. These actors exhibited different categories of power and authority as a strategy for addressing vaccine hesitancy.

### Structural power boosted accountability in health system

Structural Power is wielded through the use of an actor's positions in the structures of society to shape the thinking or actions of others. In India, structural power was leveraged to boost political ownership of the GPEI and program accountability. The polio program could not have been implemented as planned without buy-in from authorities in each state and district. KIIs described the role of structural power in improving the monitoring of booth-led activities. Structural modifications, such as regular monitoring and reporting in the review meetings to the highest authorities, undertaken at the district level, and a district task force has been created led by the district magistrate, brought greater accountability. These review meetings discussed about microplanning, vaccine shortage, adverse event following immunization. Two key informants described this process:

“*If you talk about Bihar, for example, Chief Minister used to review every month & he used to ask district magistrates directly in every meeting about the program,” KII, WHO India*.

“*District magistrate, chief medical officer questions by the monitoring reports that so many vaccination centers are not having the vaccine, AEFI reports,” KII, Stakeholder, Research Institution, India*.

Through structural power, authorities were able to regulate the behaviors of private service providers create transparency in an otherwise opaque system, and establish roles and responsibility.

“*At the district level, there were many advocacies done by the NPSP people, as I said they were in every district, I mean they were not physically sitting in every district because we did not have 600 people for the 600 districts in India. We started with 50 Surveillance Medical Officers (SMO), who would have multiple districts to their responsibility. They were able to make sure that the District Magistrates, the Chief Medical Officers, and the Political Leadership in the Districts were aligned, focused, and were moving in the right direction. So that the program was managed at the political level,” KII, Stakeholder, Partner Institution, India*.

### Moral power (power of religious leaders and institutions)

Moral Power is the degree to which an actor by virtue of his or her perceived moral stature is able to persuade others to adopt a particular belief or take a particular course of action. During the campaign, many members of the Muslim community were hesitant to take the polio vaccine. KIIs described two specific rumors: The (1) that polio drops are anti-fertility vaccines that can cause impotence and (2) that the polio vaccine has been grown in the kidney of pigs, which is considered as “*Haram*” or forbidden in the religion. These rumors contributed to vaccine hesitancy and refusal of polio drops. Many members of these communities were also based in remote areas were little or no access to mass media and communications efforts. To address this challenge, GPEI deployed the “undeserved strategy” which targeted communications efforts and advocacy initiatives to reach religious leaders in the community. These leaders were able to yield moral power to influence community members *via* faith-based teachings.

“*We also had a strategy, which we call the underserved strategy, and this involved mobilization with religious leaders and leaders from different cast groups, so we did like real-time segregation by religion and cast optimizing communities which were rejecting the vaccine and then went out, identified there the most influential people at the community level. We sat with them for a long time, however long it took up to get done that the vaccine is safe, and it is their responsibility that the community leaders, religious leaders allowed the vaccine to be accepted.” KII, Communication Partner, India*.

“*People were very enthusiastic, I would say. We didn't go and tell them that look; we are going to implement the underserved strategy here. That was only for us; we brought in their own people to explain to them. That was only part of our strategy, I mean, seeing me going there and talking to the Muslim family that you should accept compared to local mosque priests. So, we looked at the local religious leaders and educational leaders there and used them to keep the same messages we would like to give, so they would accept those more they would accept us.” KII, NGO, India*.

One communication partner described religious leaders as critical human resources for the campaign:

“*The other human resource used was religious leaders. The role of religious leaders in breaking the resistance was effortless. So, if any program has this resistance problem, one can learn from this polio program by engaging religious leaders.” KII, Communication Partner, India*.

In addition to religious advocacy, the GPEI also collaborated with secular, progressive academic institutions based in Muslim communities. The hope was that these institutions could liaise with religious institutions and create a favorable climate for the polio program.

“*There was a lot of homework done behind this whole exercise. If they are going to a Dargah “shrine of saint where people go for worship,” first they meet with some management of the Dargah and convince with him. Then only he must have allowed, and the vaccination at that particular point means that Dargah authorities are in favor of vaccination. That is why the people who are resisting in the communities are getting the children vaccinated here because there are a lot of things into it and the confidence, heading at those particular points that means the communities are in favor of vaccination and it's a national levelly dealt with it.” KII, NGO, India*.

This represents an expression of institutional power, designed to yield different institutions to influence people toward vaccine acceptance. As education institutions brought social change to their communities, these actors were further able to influence decision-making. Renowned Muslim universities exercised their power, partnered with religious institutions including Mosques and Madrasas, and collaboratively advocated for polio vaccine acceptance.

“*The big Muslim Universities like Aligarh Muslim University, Jamia Humdard University, Jamia Islamia University, etc., we brought them in, and they would be the ones advocating them for the vaccine. We brought in the local Muslim leadership into it. We had the Mosque; we had the Madarsas involved, you know there was a lot of things were done as a part of the strategy.” KII, Stakeholder, Partner Institution, India*.

### Power of experts and local influencer

Technical experts including researchers and medical professionals from both public and private sectors, joined the polio eradication movement. Voluntary organizations of doctor's Indian medical association and Delhi medical association committed to the cause, made speeches, held discussions, conducted research, wrote articles to influence the community about the advantages of taking polio vaccine, and debunked certain myths. These experts leveraged their technical skills to exert a power and influence based on their academic credentials.

“*In clinical or health programs, many voices are important. Right from a medical college professor to the Pediatrician, what they speak or what they don't speak, people listen to them. Because there is a whole influencer community in public health. Many times, we think we know everything. We can do everything. No… No… There is a tremendous role of the powerful influencer like the Indian Medical Association (IMA). They are the leaders in that space. You can't just ignore them. So, they were also involved.” KII, Stakeholder, Research Institution, India*.

In addition to well-educated and highly credentialed experts, volunteer health workers played a critical role in the success of the polio program in India. Due to gender norms, roles, and power relations, many house-to-house campaigns were implemented by female volunteer vaccinators, who were able to access homes than men vaccinators could not.

“*Earlier, we thought we could have volunteers of both genders, but we had to take female volunteers, so they could have access to houses so they could talk to family members. So that was something we had to categorize. And so as for female vaccinators also, as they have to go inside the house to give the vaccine.” KII, NGO, India*.

Female workers were able to access women-only spaces, and collaborate with women in the community to influence vaccination decisions.

“*We ensured that one every vaccination team that comprised two or three people, there was at least one female vaccinator. It was an important change because the community would not accept male workers entering their houses and vaccinating their children. We also ensured that there was a local community volunteer that was working with the vaccination team so that they could recognize this person from their community, and their acceptance would be higher.” KII, Stakeholder, Partner Institution, India*.

“*Female participation little bit has increased. Not in terms of empowering is a big word, but since this was largely a female-centric program, what happened that you have seen polio program, one female in every team has been made in Bihar essential. Without a female worker, no team will be complete. Female workers used to go inside the house and approach the mothers, talk to the mothers, and something talking about why polio is needed and other health issues. So, through this approach, they have made enroots to the family and then try to interact with the mothers or the female member in the household. So, it may have some inter-generational kind of gradually they are aware of this kind of thing (Polio Vaccination) and health programs and empowering and those things must have happened.” KII, Stakeholder, Partner Institution, India*.

### Discursive power

Discursive norms are socially accepted community tools that shape and limit what is said and done, what is say-able, what is do-able, how it is do-able, and what is truth or knowledge Actors in the polio program exercised discursive power through strategic communication to influence social decisions.

“*Organizations such as NCC, Milk distribution, and common shopkeepers, held in their way to contribute to eradicating polio. It appeared very complex; how did one work for polio eradication,” If somebody is selling milk in a packet, they put it on their packets that “Bache ko do boond pilaayiye.” If somebody is selling soft drinks, they write on their packets a similar message. Similarly, Indian railway put it on their tickets in which they also said eradicate polio from the country, so constantly messaging about eradicating the polio disease was communicated through different channels.” KII, NGO Partner, India*.

Government public service announcements featured a renowned actor, Amitabh Bachchan, who became an authoritative face. He used his discursive power to connect with people directly and support polio eradication messaging.

“*Mr. Amitabh Bachchan first came out with his public service announcements, and there was a huge spike in the number of people coming to the polio booths, and when you ask these people why they have come for polio drops, they would say that because Amitabh Bachchan told them to come,” KII Academic Institution, India*.

### Network power

One of the successful strategies of the polio eradication program was SM Net that leveraged network power. The polio program in India also employed a social mobilization network (SM.Net) strategy designed to build trust in the community using personal relationships, repeated interaction, and shared identities in the form of community influencers. A community influencer was a community member who had social, political, and economic influence to exert social pressure to motivate the resistant individual and change them to become vaccine acceptors. During the booth days, young children were engaged in spreading awareness and bringing mothers to polio booths. These influencers (community mobilization coordinators) exercised their power to work with other influential people to promote vaccination campaigns not just in this program but also in other programs.

“*One significant thing is from the social mobilization network's side was that we involved these influential peoples from the people's side that were a big breakthrough as now they were involved in other programs also not only just polio.” KII Communication Partner, India*.

“*We work through little children as mobilizers called them as “Bulawa tolies,” they would form a little gang and whoever brought children to the booth would get prizes like that, as mobilizers.” KII, NGO, India*.

**Figure 2:**
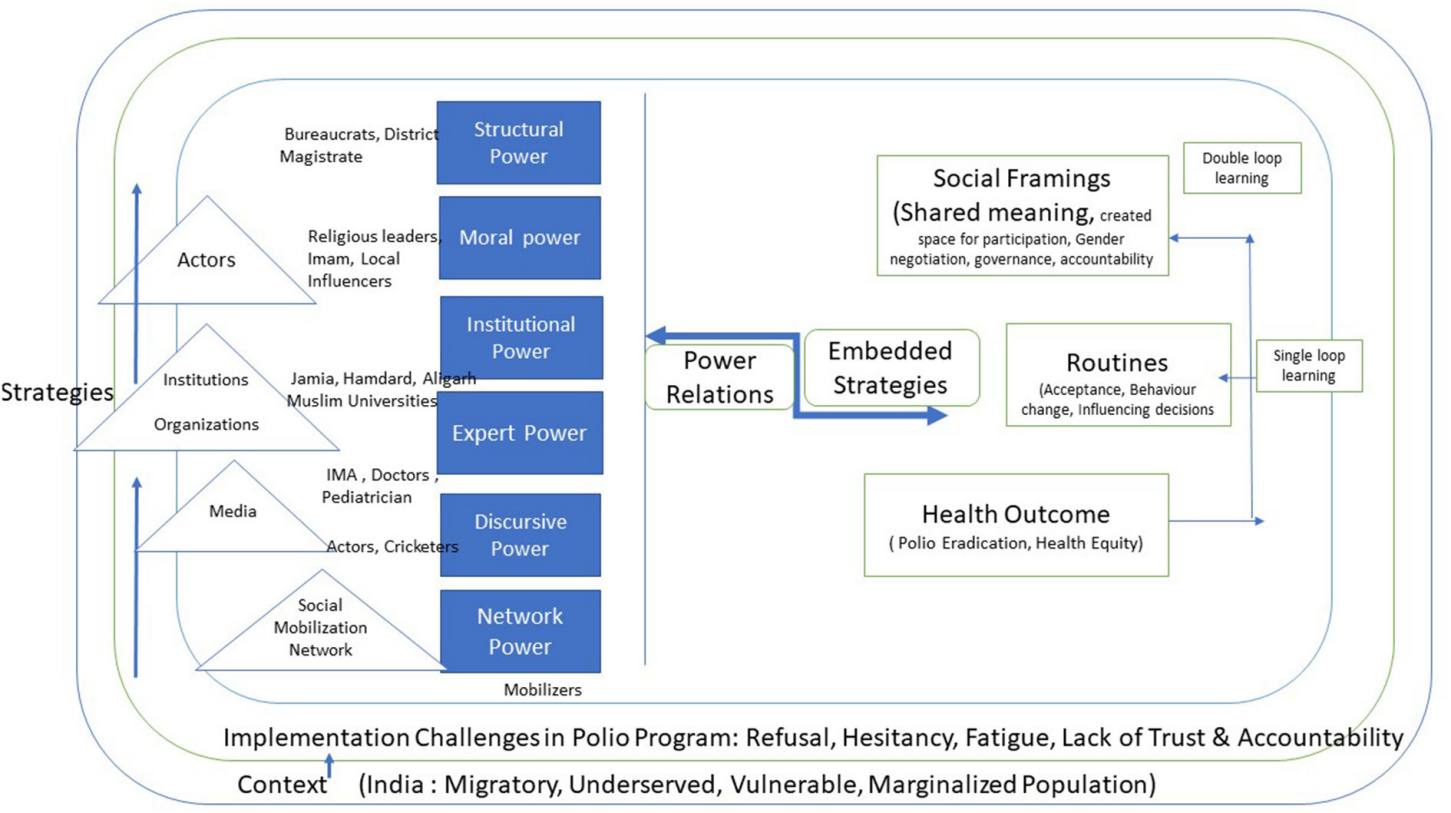
Framework of Power relation, Social learning leads to health outcomes.

We have tried to develop a framework of Power relation by analyzing moon's categories of power with respect to Polio eradication program in India (Figure 2). This framework explains the utilization of power and relation as a part of strategies to improve the social learning and improve health outcomes.

## Discussion

Evidence from the study showed that power is related to implementation challenges and can be yielded throughout the implementation process, particularly in strategy development and deployment. Our research sought to identify implementation challenges associated with one of the most extensive global health programs using CFIR. We found that GPEI implementors in India faced challenges implementing polio eradication activities at various levels and faced external challenges including vaccine hesitancy, community resistance and pervasive inequities. Other challenges, more internal to the program included workforce shortages and the GPEI's impact on other programs. Each of these demonstrate examples of groups yielding power that in some way contributed to challenges. For example, the healthcare workforce shortage was due in part to systemic factors such as low pay, long hours, heavy penalties, and few rewards. Members of the workforce were able to yield their limited power by refusing to work. GPEI's overwhelming power in the country contributed to positive changes in health policy and systems, but also stalled other health programs that became lower priority. Power can exist across the CFIR domains and is an important construct that should be considered within IR (20).

In this paper, we aimed to demonstrate how the GPEI's multipronged strategy of getting buy-in from key actors and their power dynamics to were used to persuade and influence decision-making for polio vaccination. Actors who exerted power derived it from technical expertise, political and bureaucratic position and influence, and forms of cultural capital and power gained from the title, education, and knowledge (1). We found that power is exercised by global health actors to question the public accountability of those with power, and to advocate for more inclusive agenda setting. Governments used structural power to regulate the behavior of private actors in their territories due to the state-based nature of the international system and the polio program used structural power to improve the accountability of the program.

While power is not a finite resource, it can be used, shared, or created by actors and networks in multiple ways. Power is inseparable from the development of accepted knowledge systems that power/knowledge is articulated through discursive norms (21). Those people who seem to be powerless possess invisible power that can control an individual's decision. Global health is a complex adaptive system in which many autonomous actors continuously interact and adapt producing a system of different characters (22). Furthermore, actors wield different types of power as they pursue their goals and protect their interests in the global arena. The combination of many actors simultaneously wielding various kinds of power in a complex adaptive system means that outcomes are difficult to predict or control, causality is challenging to trace or establish, and power can be difficult to discern or analyze.

At the same time, this complexity may also create opportunities for less powerful actors to wield influence (23). For example, one critical stakeholder that emerged in GPEI was the female community mobilizers who were able to enter spaces that men could not. This increased the program's access to women and girls in more conservative communities which was critical to GPEI's success. However, while female community mobilizers had increased power to influence change in communities, they still faced many challenges and were not always adequately supported by the program, facing safety issues in the home and in the communities they served. Without consideration of gender, global health programs may fail to meet their required targets, or the targets they do meet are likely to reinforce gender inequity (20).

These power categories are not mutually exclusive and may be deeply interconnected with each other. The exercise of one type of power can be conditional upon another. For example, those actors identified with moral power also use discursive power to influence the solutions that policymakers may adopt (through institutional power) to address the problem. The kind of expert knowledge considered authoritative in debates over such solutions is likely to reflect underlying distributions of economic, discursive, and network power. We have also observed Bourdieu's concept of power as social capital; one type of power can be fungible and can be transformed into another. Social capital enables a person to exert power on a group or individual who mobilize resources (24). Power is inseparable from the development of knowledge systems (“power-knowledge”) and is manifested in the creation and acceptance of truths within society, which shape and limit discourse and behavior.

Stakeholder analysis is an important activity conducted in many IR studies (25). But this analysis process can also be an expression of power. That is, who decides on the “key stakeholders” may be driven by existing norms, power, and privilege. Further, the conduct of stakeholder activities such as project planning convenings, may not account for existing power dynamics that place already marginalized groups in positions with less power to shape IR decisions. The epistemic injustice framework has been used to describe unfair knowledge practices in global health where knowledge held by people who belong to marginalized groups is afforded less credibility; this is perpetuated by complex systems that exclude these groups from knowledge generation, use, and dissemination (26). IR frameworks should account for these complexities and support IR teams to generate strategies that help identify and address power imbalances through inclusive practices and throughout the IR process.

## Strengths and limitations

STRIPE used a systematic approach for obtaining the data from the complex global health initiative. This systematic approach maps individuals involved in a complex initiative across operational levels and contexts, ensuring that perspectives are collected from actors who have a wide range of experiences (16). Our research tried to identify implementation challenges associated with one of the most extensive global health programs using CFIR. This framework is composed of 39 constructs related to effective implementation. This construct range is mainly organized into five domains: Intervention Characteristic; Outer Setting; Inner Setting; Characteristics of Individuals; and Process Like CCM (27) and PRISM (28). The presence or absence of CFIR constructs can explain “why” implementation was or was not successful. We found that implementors faced challenges in implementing polio eradication activities at various levels, faced external challenges in the form of vaccine hesitancy, community resistance, and social challenges in the form of inequities.

One of the limitations of the study was we did not address “Power” in our original KII guide, so, we might have missed nuance in the analysis.

## Conclusion

Our study demonstrated the role of power in IR and considered how power could be used as a variable within existing IR models and frameworks. Recognizing how and from where individuals, organizations and networks derive their power sharpens our understanding of how and why power flows in particular directions or accumulates with certain groups. This understanding can also facilitate awareness of how those sources of power are distributed unevenly which may be used to improve equity in health policy and systems (1). A well-developed theory enables knowledge to emerge out of seeming chaos, providing a common language for studying implementation phenomena and guiding the actual practice of implementation (29). To design and implement effective health programs, policymakers must consider programs within the context of known health systems dynamics, path dependency, and interconnectedness and power plays a key role. Further research is needed from a range of stakeholders, particularly those from LMICs and members of marginalized groups, to more clearly understand the role of power in the design of implementation strategies for global programs.

## Data availability statement

The original contributions presented in the study are included in the article/supplementary material, further inquiries can be directed to the corresponding author.

## Ethics statement

The studies involving human participants were reviewed and approved by Institution Review Board, IIHMR University, Jaipur. The patients/participants provided their written informed consent to participate in this study.

## Author contributions

SDG conceived the idea of paper, contributed to the data analysis, interpretation, and co-authored the initial draft of the paper. PM and NS were responsible for data collection. PM conducted the data analysis, contributed to the data interpretation, and authorship of subsequent draft of the paper. AK conducted the necessary edits and provided expert guidance on the manuscript. PM, AK, SDG, DM, and NS contributed to the data acquisition and provided the review of the paper for the intellectual content. All authors have read and approved the final manuscript.

## Funding

This work was supported by the Bill and Melinda Gates Foundation Project-Teaching Global Health Leaders About the Lessons Learned from Polio Eradication under its Grant Number OPP1178578 as a part of Synthesis and Translation of Research and Innovations from Polio Eradication—STRIPE (https://stripe.jhu.edu) Academic Consortium led by Johns Hopkins Bloomberg School of Public Health.

## Conflict of interest

The authors declare that the research was conducted in the absence of any commercial or financial relationships that could be construed as a potential conflict of interest.

## Publisher's note

All claims expressed in this article are solely those of the authors and do not necessarily represent those of their affiliated organizations, or those of the publisher, the editors and the reviewers. Any product that may be evaluated in this article, or claim that may be made by its manufacturer, is not guaranteed or endorsed by the publisher.
